# Altered Synthesis of Cartilage-Specific Proteoglycans by Mutant Human Cartilage Oligomeric Matrix Protein

**DOI:** 10.4055/cios.2009.1.4.181

**Published:** 2009-11-25

**Authors:** Yoon Hae Kwak, Jae Young Roh, Ki Seok Lee, Hui Wan Park, Hyun Woo Kim

**Affiliations:** Department of Orthopaedic Surgery, Hallym University Sacred Heart Hospital, Anyang, Korea.; * Department of Orthopaedic Surgery, Konyang University Hospital, Daejeon, Korea.; † Department of Orthopaedic Surgery, Severance Children's Hospital, Yonsei University College of Medicine, Seoul, Korea.

**Keywords:** Mutant COMP, Extracellular matrix, Pseudoachondroplasia

## Abstract

**Background:**

The mechanism by which mutant cartilage oligomeric matrix protein (COMP) induces a pseudoachondroplasia phenotype remains unknown, and the reason why a mutation of a minor protein of the growth plate cartilage causes total disruption of endochondral bone formation has not yet been determined. The current study was performed to investigate the effects of mutated COMP on the synthesis of the cartilage-specific major matrix proteins of Swarm rat chondrosarcoma chondrocytes.

**Methods:**

The Swarm rat chondrosarcoma chondrocytes transfected with a chimeric construct, which consisted of a mutant gene of human COMP and an amino acid FLAG tag sequence, were cultured in agarose gel. Formation of extracellular proteoglycan and type-II collagen by the cells was evaluated by immunohistochemical staining and measuring the ^35^S-sulfate incorporation.

**Results:**

No difference was observed for the detection of type-II collagen among the cell lines expressing mutant COMP and the control cell lines. Histochemical staining of sulfated proteoglycans with safranin-O showed that lesser amounts of proteoglycans were incorporated into the extracellular matrix of the chondrocytes transfected with the mutant gene. ^35^S-sulfate incorporation into the cell/matrix fractions demonstrated markedly lower radiolabel incorporation, as compared to that of the control cells.

**Conclusions:**

Mutation of COMP has an important impact on the processing of proteoglycans, rather than type-II collagen, in the three-dimensional culture of Swarm rat chondrosarcoma chondrocytes.

Longitudinal bone growth is controlled by endochondral ossification and this requires smooth orchestration of chondrocytes proliferation, cellular differentiation and the formation of the extracellular matrix (ECM) in the growth plate. Mutations in many different ECM protein genes can interfere with this orchestration and disrupt normal skeletal growth.^1^-3) Among them, cartilage oligomeric matrix protein (COMP) has been identified as the abnormal protein in two types of human autosomal dominant skeletal dysplasias: pseudoachondroplasia (PSACH) and multiple epiphyseal dysplasia.^4^,5) The tissue pathology of the growth plate cartilage is striking in patients with PSACH. Electron microscopy has shown marked dilatation of the rough endoplasmic reticulum (rER) of those chondrocytes with COMP retained in them.^6^-8)

COMP is a 524-kDa homopentameric extracellular matrix protein that consists of 737 amino acid monomeric units linked through the interchain disulfide bonds at Csy49 and Cys52. The single gene copy is coded from 19 exons that have strong sequence homology within exons 4-19 to four other known thrombospondin genes (exons 11-22).9) Most of the mutations in PSACH have been identified in the highly conserved type-3 repeat calcium-binding domain that is encoded by exons 13C to 18A. Previous studies have shown that the COMP that harbors mutations within the type-3 repeat binds to half the number of Ca^2+^ ions compared to the wild type protein,^9^,10) and that the wild and mutant type proteins show different conformations in the presence of calcium.9) These findings suggest that mutation specifically affects COMP processing by impeding the normal protein folding that is needed for its secretion from the rER.

As a minor extracellular matrix protein, COMP was originally identified in cartilage^11^,12) and it has been since been identified in the cells of ligaments and tendons.^8^,13) According to the electron microscopic findings, retention of COMP in patients with PSACH is specific to chondrocytes. However, a monolayer culture of PSACH chondrocytes has shown cellular dedifferentiation to a fibroblast-like phenotype and disappearance of the intracellular retention of COMP.14) Furthermore, the inability to distinguish mutant human COMP (hCOMP) from the wild type being expressed in PSACH chondrocytes, and the lack of the expression of mutant hCOMP to generate a PSACH phenotype in any cells other than chondrocytes have hampered the studies on monitoring the processing of mutant COMP and its effect on the formation of other ECM molecules.

The current study was undertaken to investigate the effects of a mutant hCOMP on the secretion of cartilage-specific proteins such as proteoglycans and type-II collagen. A three-dimensional culture of chondrocytes transfected with a chimeric construct containing a mutant hCOMP coupled with a *FLAG* tag sequence was utilized, and this readily permitted selective monitoring of the mutant hCOMP_*FLAG*_ during the processing of rat endogenous COMP (rCOMP).

## METHODS

### Three-dimensional Agarose Cell Cultures

**Fig. 1:**
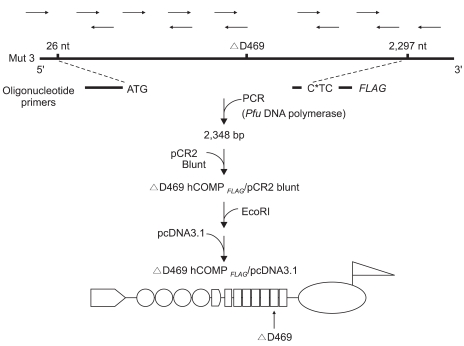
Construction of a mutant hCOMP_*FLAG*_ chimeric protein. A primer set consisting of an oligonucleotide sequence that encompasses the transcription start site and an oligonucleotide containing the 3' end of COMP with a mutation of the stop codon, plus a sequence that encodes for the 8 amino acid *FLAG* epitope was used to generate a 2348 nucleotide PCR product from the clone Mut3 that encodes for PSACH-linked ΔD469 hCOMP. The DNA fragment was inserted into the pcDNA 3.1 expression vector following ligation into the pCR2 vector blunt to obtain the *Eco* RI DNA restriction enzyme sites.

We used three clones that came about from the development of stable transformed long-term culture (LTC) cell lines (a gift from Dr. J. W. Stevens of The University of Iowa).^6^,^9^,^14^-16) The DNA vector constructs were originally transfected into the Swarm rat chondrosarcoma LTC cells16) with using either lipofectin (Life Technologies, Grand Island, NY, USA) or SuperFect (Qiagen Inc, Valencia, CA, USA) per the manufacturer's procedure. Clone "C415" consisted of a DNA construct that express the PSACH-linked mutant COMP (deletion of aspartic acid 469) and an 8 amino acid *FLAG* tag sequence that was utilized for the immunodetection of the expressed mutant hCOMP_*FLAG*_ with performing western blotting in a previous study (Fig. 1).15) Clone "C422", which contained an antisense sequence of the mutant hCOMP, was used as a transfectant negative control, and the LTC cells of the Swarm rat chondrosarcoma were used as a control cell line.

Three cell lines were individually cast in dialysis tubes (4 mm in diameter) at 5 × 10^6^ cells/ml in a 1% low melt agarose-complete growth medium cell culture suspension. The castings were maintained in complete growth medium16) that consisted of Dulbecco's Modified Eagle's Medium (4.5 gm glucose per liter), 12% heat inactivated fetal bovine serum, 25 mM N-2-hydroxyethylpiperazine-N'-2-ethane sulfonic acid, ascorbic acid 50 µg/ml and gentamycin 50 µg/ml. The growth medium was changed daily.

### ^35^S Incorporation Assay

Three sections of the castings (0.5-1 cm in length) were removed per cell line and these were cultured with ^35^S-H_2_SO_4_ at 50 µCi/ml in growth medium for 24 hours prior to harvesting at days 1, 4, 7, 14 and 28. Following washing and removing the unincorporated radiolabel, the castings were frozen in Tissue Freezing Medium (Triangle Biomedical Science, Durham, NC, USA). Thirty 10 µm sections cut on a cryomicrotome were taken from each casting (3 per cell line, per time point) with sections 1-5, 11-15 and 21-25 being pooled in microfuge tubes. The pooled sections were melted at 72℃ in 500 µl of papain solution (- 17 units/mg protein from papaya latex, Sigma-Aldrich, St Louis, MO, USA) at 0.1 mg/ml in 0.1 M sodium acetate (pH 6.5) and 5 mM mannitol. Sodium azide (0.025%, w/v) was added and then this was incubated at 60℃ for 24 hours. Digest was applied to a Sepharose G-50 column (0.7 × 14 cm), equilibrated in papain digestion buffer, void column volume, devoid of unincorporated radiolabel, and was collected. The amount of radiolabel was determined in a liquid scintillation counter (Model LS380; Beckman, Fullerton, CA, USA) following the addition of 4 ml of Bio-Safe II counting cocktail (Research Products International Corp., Mount Prospect, IL, USA). Statistical analysis was performed by ANOVA.

### Immunohistochemical Studies

To randomly analyze the sections, every 10th section was singularly analyzed starting with the first collected section, and each analysis was performed in triplicate. A series of sections was fixed in 10% neutral formalin and embedded in paraffin per the usual method. Five-micron sections were placed on gelatin-coated slides, deparaffinized with xylene and then hydrated with in a graded series of ethanol solutions. The sections were washed with 20 mM sodium phosphate (pH 7.4) and 150 mM NaCl and the sections were treated with Target Unmasking Fluid (Signet Laboratories, Dedham, MA, USA) at 70℃ for 20 minutes. Following two washes with 20 mM sodium phosphate (pH 7.4) and 0.15 M sodium chloride, the sections were treated with chondroitin ABC lyase (Seikagaku America, Rockville, MD, USA) at 0.25 units/ml and *Streptomyces* hyaluronidase (Calbiochem, La Jolla, CA, USA) at 0.625 units/ml in 0.1 M Tris-HCl and 0.1 M sodium acetate (pH 7.2) for 90 minutes at 37℃.

The sections were washed three times with 20 mM sodium phosphate (pH 7.4) and 150 mM NaCl for 5 minutes each time, and the sections were then treated with 0.3% hydrogen peroxide in methanol for 30 minutes at room temperature, and then they were washed three times for 5 minutes each time. The samples were blocked with 10% horse serum (for M2 antibody, Eastman Kodak Company, Scientific Imaging System, New Haven, CT, USA), 10% goat serum (for anti-ratCOMP antibody), 1% bovine serum albumin and 0.1% Tween-20 in 20 mM sodium phosphate (pH 7.4) and 150 mM NaCl at room temperature for 30 minutes. The *FLAG* epitope containing species were detected with M2 antibody at 1 µg/ml, while the total COMP (rat and human) was detected with a rabbit polyclonal antibody generated against the 8 amino acid carboxyl terminus of rCOMP, which has 62.5% homology to the human COMP carboxyl terminus sequence.17) The samples were washed three times with 20 mM sodium phosphate (pH 7.4) and 150 mM NaCl for 5 minutes each time. A series of sections was treated with murine monoclonal antibodies for II-II63B (type II collagen, gift from Dr. J. W. Stevens) and M2 antibody, and this was developed with DAB substrate following the addition of biotin-conjugated anti-murine IgG secondary antibody and horseradish-conjugated streptovavidin. Additionally, some sections were processed for safranin-O staining for identifying cartilage-specific sulfated glycosaminoglycans.

## RESULTS

**Fig. 2:**
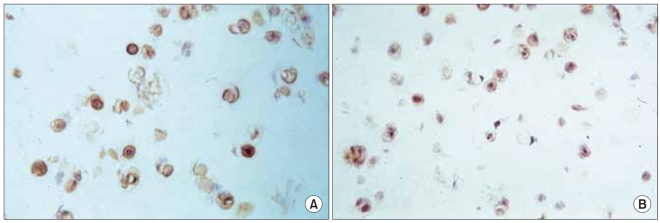
Immunohistochemical staining of type II collagen at day 14. No differences for type II collagen were observed between the expressed mutant COMP cell line (C415) (A) and that of the antisense transfectant control cell line (C422) (B) (×200).

**Fig. 3:**
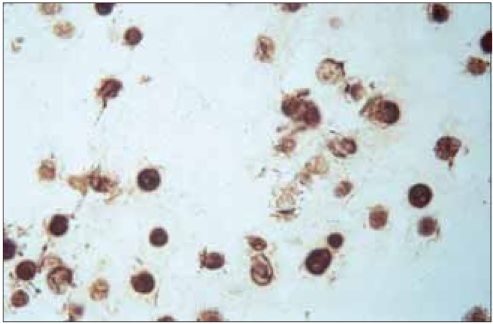
Immunohistochemical identification of human PSACH-linked mutant COMP tagged with a selective immunoreactive sequence that was distinct from that of rat COMP in the Swarm rat chondrosarcoma cells cultured for 14 days. Brown staining identifies the retention of expressed mutant COMP within the cells (×1,500).

Immunohistochemical staining of type II collagen, following the culture of the human mutant COMP cell line (C415) and the two control cell lines of C422 and LTC, showed that type II collagen was detected in all the cell lines at day 1, and an increase in staining intensity continued through day 7 with no apparent additional increase at day 14 or 28 thereafter. No differences were observed for detecting type II collagen between the expressed mutant COMP cell line and those of the control cell lines (Fig. 2). Immunodetection study of the mutant COMP-*FLAG* epitope following culture of the mutant hCOMP_*FLAG*_ cell line C415 showed that mutant hCOMP_*FLAG*_ was detected intracellularly at day 1 with a slight increase in intensity at day 4 (Fig. 3). *FLAG* positive staining was also detected in what appears to be the pericellular matrix of the cells, suggesting that a portion of the mutant COMP was secreted by the cells.

**Fig. 4:**
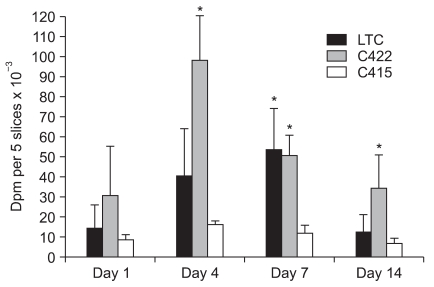
^35^S-sulfate incorporation into the cell/matrix fractions of the cell lines cultured in 1% agarose for varying times (Days 1, 4, 7, and 14). The C415 cells expressing the mutant hCOMP_*FLAG*_ , demonstrated markedly lower radiolabel incorporation compared to the C422 mutant hCOMP_*FLAG*_ antisense control cells and the LTC cells. An asterisk indicates statistically significant difference at *p* < 0.05, as compared to the C415 cells.

^35^S-sulfate incorporation at various times showed that identifiable newly formed proteoglycans from all the cultured cells were seen through day 14 with the greatest amount being synthesized at days 4 and 7 (Fig. 4). For the four time points, the C415 cells incorporated from 8.9 × 10^3^ ± 2.3 × 10^3^ to 16.5 × 10^3^ ± 1.6 × 10^3^ dpm per 5 slices while the LTC cells incorporated from 12.2 × 10^3^ ± 9.2 × 10^3^ to 53.4 × 10^3^ ± 0.1 × 10^3^ dpm per 5 slices and the C422 cells incorporated from 31.1 × 10^3^ ± 24.3 × 10^3^ to 98.6 × 10^3^ ± 22.4 × 10^3^ dpm per 5 slices. The C415 cells that expressed the mutant hCOMP_*FLAG*_ universally incorporated less radiolabeled sulfate than that of the C422 and LTC control cell lines. The C415 cells incorporated less radiolabeled sulfate compared to the hCOMP antisense C422 clone at days 4, 7 and 14 (17-24% less) and the LTC cells at day 7 (22% less) (*p* < 0.05). Undetected amounts of radiolabeled sulfate were incorporated into the cells at the 28 day time point (data not shown), suggesting a cell to ECM homeostatic equilibrium had been reached, as was reflected by a minimal degree of newly synthesized proteoglycans.

**Fig. 5:**
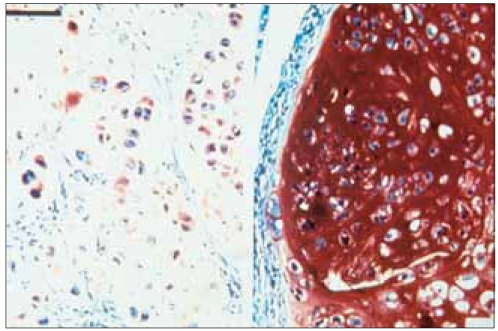
Histochemical staining of sulfated proteoglycans with safranin-O. Lesser amounts of proteoglycans are incorporated into the ECM in the C415 cell line (left panel) compared to the C422 cell line (right panel), suggesting the expression of the mutant COMP is altering the deposition of aggrecan in the ECM, as is similarly seen in PSACH patients (×200).

Histochemical staining of the sulfated proteoglycans with safranin-O showed that lesser amounts of proteoglycans were incorporated into the extracellular matrix of the cells containing the mutant hCOMP_*FLAG*_ transgene (Fig. 5), suggesting that the expression of the mutant COMP reduces the deposition of aggrecan in the extracellular matrix.

## DISCUSSION

In individuals with PSASH, their dramatically reduced limb lengthening appears to the result of an altered expansion of the extracellular matrix and longitudinal growth of the chondrocytes prior to ossification.^17^-20) Lamellar inclusion of the rER in the growth plate chondrocytes is the cytochemical hallmark for the PSACH phenotype, linking an endoplasmic reticulum storage disorder with the osteochondrodysplasia. A PSACH canine model suggested that abatement of the cumulative vertical growth of the chondrocytes is caused by 1) altered extracellular matrix constraints for horizontal growth and 2) uncoupling of the endochondral and perichondral growth that causes metaphyseal flaring.21)

COMP is selectively expressed in the cells of cartilage, tendon, ligament and synovium. In the growth plate cartilage of PSACH individuals, COMP is localized to the electron-lucent lamellae of the rER, but the rERs of the tendon and ligament tissues are unaltered. Normal growth and development occurs in COMP gene knockout mice that do not synthesize COMP, demonstrating that a mutant COMP, not the absence of COMP, is required for the PSACH phenotype.22) However, the mechanism by which mutant COMP induces a PSACH phenotype remains unknown, and the reason why a mutation of a minor protein of the growth plate cartilage causes total disruption of endochondral bone formation remains to be determined. The altered conformation of COMP induced by mutation has been proposed for the possible mechanism for COMP not being properly processed from the rER.14) In patients with PSACH, COMP is retained intracellularly, but no direct evidence has demonstrated that a mutant COMP itself is responsible for its retention. Moreover, subtle amino acid differences of the mutant COMP, as compared with the wild type, preclude monitoring the expression of the individual COMP species.

The use of a PSACH-like cell culture system with unlimited numbers of cells of the chondrosarcoma cell line permitted us to perform the manipulative experiments, which are impossible when using human tissue or isolated cells. Furthermore, the reason why we used a three-dimensional culture of chondrosarcoma cells is that the cartilage formed during the monolayer culture of chondrocytes becomes increasingly fibrous in nature when the cells are cultured for extended periods of time.23)

The extracellular matrix of the growth plate cartilage functions as a template for future trabecular bone, and this consists of tissue fluids and a framework of structural macromolecules such as type-II collagen, proteoglycans and non-collagenous proteins. The current study demonstrated that the expression of a PSACH-linked mutant COMP in a rat chondrosarcoma cell line alters extracellular matrix formation with regard to incorporating proteoglycan into the extracellular matrix. This can be explained by the effects of mutant COMP on the post-translational processing of proteoglycans and the addition of the glycosaminoglycan chains in the Golgi apparatus. However, we think that this is not a general defect in the protein processing. For example, the secretion of cartilage-specific type-II collagen was found to be unaltered for its pericellular site distribution relative to the control cell lines.

Our data imply that the defect affects a subset of extracellular matrix molecules that includes proteoglycans, and that mutant COMP selectively alters the incorporation of proteoglycan molecules into the ECM. Secretory proteins follow the process of first being translated, followed by folding and assembly within the cisterne with the aid of chaperone proteins, and then the proteins are exported to the Golgi via rER vesicles for their secretion. Similar chaperone proteins are used in common pathways. Thyroglobulin and thrombospondin are proteins that undergo oligomerization, and they are both associated with a complex of multiple molecular chaperones composed of BiP, grp94, Erp72 and grp170 proteins.^24^,25) COMP and proteoglycan are thought to potentially share a similar secretory pathway, and the introduction of a mutation in the COMP molecule causes retention of aggrecan in the rER and the resultant decreased proteoglycan secretion.

In our study, lesser amounts of proteoglycans were secreted during the establishment of molecular homeostasis of the extracellular matrix. It suggests that the matrix is being precisely orchestrated during its formation and the expression of the mutant COMP alters this. Even with extended culture periods of cells expressing the mutant COMP, the level of secreted proteoglycans did not catch up to the amount of proteoglycans synthesized by the control cells, and this suggests a link between the protein export of COMP and proteoglycans is altered from the normal situation when the mutant COMP is expressed.

Future studies deciphering the mechanism whereby mutant COMP alters the processing of aggrecan into the ECM will potentially lead to understanding the altered growth of the individuals with PSACH. Potentially, the mutant COMP is being cleared from the rER via a scavenger route that is common for the miss folded or assembly molecules being processed,^26^-28) and further studies, such as pulse-chase experiments, for identifying the fate of the mutant COMP and its turnover rate would help clarify what is occurring to the newly synthesized mutant COMP.

In conclusion, we showed that a mutation in human COMP affects the secretion of proteoglycans as well as the COMP itself, and the dramatically reduced limb lengthening in PSACH individuals appears to the result of the altered formation of sulfated proteoglycans rather than the altered formation of type-II collagen. However, it should be noted that this experiment was conducted on animal cells that were transfected with one form of a mutant human gene sequence among the variety that cause PSACH and MED, and these results are not necessarily true for the chondrocytes of PSACH patients.
